# A Case Control Study on Serum Levels of Potential Biomarkers in Male Breast Cancer Patients

**DOI:** 10.3390/ijerph18094852

**Published:** 2021-05-01

**Authors:** Kamal Eldin Ahmed Abdelsalam, Mohammed Asad, Monjid Ahmed Ibrahim Ahmed, Syed Mohammed Basheeruddin Asdaq, Yahya Mohzari, Ahmed Alrashed, NajwaJilan Alghamdi, Kholoud Nasser Alrami, Wael Ahmed Alharbi

**Affiliations:** 1 Department of Clinical Laboratory Science, College of Applied Medical Sciences, Shaqra University, Shaqra 11911, Saudi Arabia; masad@su.edu.sa (K.E.A.A.); mohammedasad@hotmail.com (M.A.); asad_asiya@yahoo.com (M.A.I.A.); 2 College of Medical Laboratory Science, Omdurman Islamic University, Omdurman 825109, Sudan; 3 Faculty of Science and Technology, Al-Neelain University, Khartoum 11121, Sudan; 4 Department of Pharmacy Practice, College of Pharmacy, AlMaarefa University, Dariyah, Riyadh 13713, Saudi Arabia; 5 Clinical Pharmacy Department, King Saud Medical City, Riyadh 12746, Saudi Arabia; Yali2016@hotmail.com; 6 Pharmaceutical Services Administration, Inpatient Department, Main Hospital, KFMC, Riyadh 11564, Saudi Arabia; emadasdaq@gmail.com; 7 Department of Pharmacy, Adham General Hospital, Adham 28653, Saudi Arabia; mhospital1920@gmail.com; 8 Pharmaceutical Services Department, King Fahad Medical City, Riyadh 11564, Saudi Arabia; emadfaiqa@gmail.com (K.N.A.); abstracts@mcst.edu.sa (W.A.A.)

**Keywords:** dopamine, epinephrine, GABA, ghrelin, melatonin, serotonin

## Abstract

The global incidence of breast cancer among men is steadily growing. Despite this, compared to female breast cancer patients, there are very few studies on biomarkers in male breast cancer patients. A cross-sectional case control study was carried out to determine the serum levels of melatonin, ghrelin, dopamine, serotonin, epinephrine, and GABA in male breast cancer. All the recruited patients were obese, old, and had recently been diagnosed with the disease. They had not received any treatment for the cancer until the time of the study. Melatonin and epinephrine serum levels were significantly higher in breast cancer patients compared to their age-matched controls, whereas ghrelin, dopamine, GABA, and serotonin serum levels were lower in patients compared to the control group. The serum levels of most of the studied biomarkers in male breast cancer patients were similar to those observed in female breast cancer patients, except for serum melatonin levels.

## 1. Introduction

About 1% of all breast cancer cases occur in men, and its incidence has increased by 26% in the last three decades [1]. The risk of developing breast cancer is about 70-fold less common in black men compared to black women, which is slightly more compared to white men in whom it is less than 100-fold less common compared to white women [2]. Breast cancer among men is rare; therefore, there is limited research on this topic, although it is known that the survival rate among male breast cancer patients is less compared to female breast cancer patients [3]. Risk factors for the development of male breast cancer include genetic causes that encompass *BRCA2* mutations and environmental influences, such as advanced age, antiandrogen treatment, radiation therapy and hormonal imbalances (elevated estrogen levels and reduced testosterone levels) [4]. The presence of estrogen receptors or progesterone receptors in breast cancer cells is routinely used to identify if the cancer will respond to hormonal therapy [4]. Although several risk factors are known, it is widely accepted that various biological markers should be evaluated for better diagnoses of this disease and its associated complications.

Several biomarkers have been studied in female breast cancer patients, and each of these has been reported to help in the diagnosis of associated complications in breast cancer patients. Gamma-amino butyric acid (GABA) is a well-known inhibitory neurotransmitter in the brain cortical region [5]. Apart from its neuronal effects, GABA is known to reduce cancer, diabetes, inflammation, allergy, and oxidative stress. Furthermore, it is reported to have a role in augmenting damage to the kidney, liver, and the intestine [6]. Melatonin is a ubiquitous chemical synthesized in many tissues, including the suprachiasmatic nucleus, and is mainly responsible for regulating the light–dark cycle [7]. Moreover, the immune system, homeostasis, glucose regulation, and scavenging of oxidative radicals depends on melatonin levels [8]. It is also reported to reduce the development of several types of cancer [9]. Ghrelin is a peptide hormone released mainly by the gastric mucosa along with many other cells. It is involved mainly in the regulation of glucose metabolism [10]. Ghrelin is called a homeostatic force because it regulates the internal environment and inflammation in many organs [11]. Ghrelin is known to have a biological role in the progression of cancer [12]. GABA, melatonin, and ghrelin have been evaluated for their role in breast cancer in females [13,14,15]. Dopamine is known to have several effects, including an increase in heart function and the development of psychosis, and lack of dopamine in substantia nigra is associated with Parkinson’s disease [16]. The role of dopamine in the sensitivity of breast cancer in females to anticancer drugs has also been studied in detail [14]. Epinephrine (also called adrenaline) and serotonin (also known as 5-hydroxytryptamine) have several effects on different organ systems. Both these neurotransmitters are strongly implicated in the pathogenesis of depression, and drugs that increase their levels in the brain are used as antidepressants [17]. To determine the anxiety and depression faced by patients, epinephrine and serotonin have also been determined in female breast cancer patients [18]. The current study aimed to determine the serum levels of these biomarkers in order to assess their roles in male breast cancer patients, which are still unclear.

## 2. Materials and Methods

This was a cross-sectional case control study performed by recruiting patients attending clinics in Khartoum (Sudan) through convenience sampling. A group of 16 male patients with clinical proof and laboratory confirmation of breast cancer were recruited. All patients had symptoms suggestive of male breast cancer that included a lump in the breast, nipple turning inward, skin puckering around the nipple, and/or discharge from the nipple for a duration of six months to one year. Breast cancer patients that had undergone any type of treatment were excluded from the study. Breast cancer in all patients was confirmed by mammography and core needle biopsy. All male breast cancer patients had no indication of metastasis to lymph nodes or other body parts. The breast cancer subjects lived separate authentic, cultural backgrounds, without previous record of smoking, diabetic symptoms, hormonal health issues, hypertension, renal or hepatic medical condition, or even family history of breast cancer. The blood samples were collected from three different centers in Sudan; S.C.F Hospital, Dr. Sami A Badr (Private Oncology Clinic), and Al-Afaf Specialized Clinics between March, 2017 and December, 2019. A pre-prepared questionnaire was used to gather information about the patients and their breast cancer (such as age, family history, type of treatment, and BMI). The Ethical Committee of the Advanced Research Centre (Sudan) approved the methodology, and informed consent was obtained from all the participants.

The study control group had 16 normal healthy volunteer males matching the demographics of the recruited patients. Venous blood samples (6 mL) were withdrawn from all subjects between 8:30 a.m. and 11:00 a.m., and sera were assayed for serotonin, ghrelin, dopamine, melatonin, adrenaline, and gamma-aminobutyric acid (GABA) by an enzyme-linked immunosorbent assay (ELISA) according to the manufacturer’s protocol. Serotonin (E-EL-0033), ghrelin (E-EL-H1919), melatonin (E-EL-H2016), dopamine (E-EL-0046) and epinephrine (E-EL-0045) kits were obtained from Elabscience (Wuhan, China), while the GABA (K7012) kit was from Immundiagnostik AG (Bensheim, Germany). The estrogen receptor status was assessed by immunohistochemistry [19].

The data are expressed as the mean ± SD. Differences between groups were evaluated by using an unpaired *t*-test with *p*-value < 0.05 considered statistically significant. Furthermore, the Pearson’s correlation coefficient between different variables in the control group and study group was performed.

## 3. Results

The mean age of recruited patients was 68 years; accordingly, volunteers in the same age group were recruited as controls. The BMI in the study group was about 38.8, which indicated that patients were obese, and this was not significantly different from the control group. Significantly higher serum melatonin levels were observed in male breast cancer patients compared to the control group (*p* < 0.01). The serum levels of ghrelin, GABA and dopamine were significantly lower in male breast cancer patients compared to the controls. As expected, a significant increase in serum epinephrine levels (*p* < 0.001) and a significant decrease in serum serotonin levels (*p* < 0.001) were detected in the study group compared to the control group (Table 1).

Until the time of diagnosis, the male breast cancer patients had not received any form of treatment, whether chemotherapeutic, endocrine, or radiological. There was no metastasis, and the histological grade was either 1 (69%) or 2 (31%). About 63% of patients were estrogenic receptor-positive. All patients had palpable breast mass, while most of the patients suffered from distention in one breast. Patients did not report any pain in both breasts, except for one patient who suffered from breast pain and bone pain (5 months duration). There was puckering of the breast skin, nipple discharge, and shrinkage of the nipple. Some patients suffered from nipple ulcers and swelling in the lymph glands (underarm area). Mastectomy was performed on all of the patients. Histology reports showed that all patients had ductal carcinoma stage II, and none of them had a family history of breast cancer. None of the patients had metastases to any organ (clinically + ultrasound test). As mentioned earlier, none of the patients had undergone any chemotherapy, radiotherapy, or endocrine therapy.

The Pearson’s correlation coefficients between different variables in the control group and study group are shown in Table 2 and Table 3. There were both positive and negative correlations between the parameters. Age was significantly correlated with serum melatonin levels in the control group. However, in the study group, serum ghrelin levels and serum melatonin levels were significantly correlated with BMI and serum GABA levels, respectively.

Comparison of BMI and serum biomarkers between estrogen receptor-positive and estrogen receptor-negative breast cancer patients did not reveal any significant differences between these two groups (Table 4).

## 4. Discussion

Diagnostic factors are used in everyday practice for identifying patients with early breast cancer [8,15]. Many biomarkers have been used to classify early breast cancer patients, and several intracellular pathways, receptors, and epigenetic processes influence breast cancer survival, proliferation, and migration in females. Breast cancer in women has also been thoroughly studied, and a number of risk factors have been reported. Male breast cancer studies, on the other hand, are relatively few, owing to the lower number of cancer patients. Many biomarkers have been estimated in this study to evaluate underlying diseases and conditions in breast cancer patients, in addition to other diagnostic factors used for breast cancer diagnosis.

In the present study, patients recruited were around 68 years of age and were obese, with a BMI of around 38. Both age and obesity are known risk factors for the development of breast cancer in men [1].

Melatonin is a ubiquitous molecule that is synthesized in the pineal gland during the night, induced by darkness. Its levels are regulated by the environmental light/dark cycle via the suprachiasmatic nuclei. Apart from its involvement in the circadian rhythm, it is an effective antioxidant that is known to scavenge oxygen free radicals and increase the expression of several endogenous antioxidant enzymes [20]. It is known that melatonin is anti-estrogenic in nature and is used in the treatment of female breast cancer. Melatonin inhibits the growth of breast cancer cells by preventing angiogenesis, metastasis, and telomerase activity. By altering several signaling pathways, melatonin results in anticancer activity [9]. Additionally, melatonin also augments the therapeutic benefits of chemotherapeutic agents when administered concurrently to patients with breast cancer [20,21]. Earlier studies on the serum levels of melatonin in female breast cancer showed that patients who had lower levels of serum melatonin correlated with the prevalence of depression and anxiety. However, in the current study, significantly higher serum melatonin levels were observed in male breast cancer patients compared to their age-matched controls. This difference between male and female breast cancer patients warrants further investigation to determine the effects on sleep and whether increased serum melatonin levels augment the therapeutic benefits of cancer chemotherapeutic agents.

Ghrelin is a peptide that is known to regulate growth hormone secretion, food intake and insulin secretion, adipogenesis, and gastrointestinal motility [22,23]. The serum level of ghrelin is higher in females, and it is reported to have a role in the tumorigenesis of breast cancer; obesity, however, a known risk factor for breast cancer, is associated with low ghrelin levels. Furthermore, single nucleotide polymorphisms (SNPs) in the ghrelin gene are reported to increase the breast cancer risk in females [10,11]. An earlier report on the expression of ghrelin peptides in male breast cancer patients revealed that increased expression of this peptide reduces breast cancer risk. [24]. A significant decrease in serum ghrelin levels was observed in male breast cancer patients. This result is consistent with other reports in female breast cancer patients, that low serum ghrelin levels are observed in breast cancer patients.

One of the well-known inhibitory neurotransmitters is γ-aminobutyric acid (GABA). It is known that there is an alteration in GABAergic signaling in breast cancer cells [25]. GABA receptor stimulation is reported to decrease the proliferation of tumor cells and metastasis of breast cancer cells, signifying the potential role of GABA in increasing the life expectancy of female breast cancer patients [26,27]. A decrease in serum levels of GABA was found to increase the risk of death in female breast cancer patients, indicating that GABA is an important diagnostic factor for female breast cancer patients’ survival [5]. In the current study, the levels of GABA in breast cancer patients were about 43% lower compared to that in normal volunteers.

The role of dopamine in different diseases and disorders has been studied thoroughly. It acts through two groups of receptors: D1-like receptor (D1 and D5 dopamine receptor) and D2-like receptor (D2, D3 and D4 dopamine receptor) [28]. Thioridazine, a D2 receptor antagonist, was reported to target cancer stem cells [29]. Dopamine, through its action on the D1 receptor, is known to increase the sensitivity of drug-resistant breast cancer to sunitinib [30]. Peripheral dopamine is reported to have a role in different types of cancer through various mechanisms [31]. The serum level of dopamine has not been measured before, in either female or male breast cancer patients. A significant decrease in the study group was observed when compared to the control group.

The disturbance of estrogen to testosterone levels contributes significantly to the development of male breast cancer. Although it is widely reported that there is an increase in estrogen levels and a decrease in testosterone levels, a few studies have indicated that only estrogen levels are increased, with no change in testosterone levels [32]. In the current study, no attempt was made to determine estrogen or testosterone levels, but the estrogen receptor-positive status was determined. About 63% of male breast cancer patients were estrogen receptor-positive. A comparison of BMI and serum biomarkers between estrogen-positive and estrogen-negative patients did not show any significant difference.

Depression and anxiety are common among female breast cancer patients. Patients also experience a reduced quality of life and have to bear higher healthcare costs due to these psychiatric disturbances [33]. Increased anxiety and depression serve as risk factors for facilitating tumor growth by suppressing immunity [34]. In the present study, increased serum levels of epinephrine and reduced levels of serotonin may be due to stress and depression experienced by the male breast cancer patients.

Identification of serum levels of melatonin, ghrelin, dopamine, epinephrine, serotonin, and GABA revealed different results. Most of the serum levels of the measured biomarkers were similar to those observed in female breast cancer patients, such as melatonin, ghrelin, GABA, epinephrine, and serotonin. The serum levels of dopamine were significantly lower in male breast cancer patients compared to normal controls.

Finally, it should be emphasized that the above biomarkers cannot be used for routine breast cancer diagnosis, but their determination can reveal potential underlying disorders and diseases in male breast cancer patients.

## Figures and Tables

**Table 1:** Difference in serum biomarkers between the control group and study group.

Parameter	Control Group	Study Group
Age (years)	65.16 ± 2.8	68.32 ± 7.34
BMI	34.90 ± 1.18	38.32 ± 1.86
Melatonin (pmol/L)	51.79 ± 7.52	61.41 ± 8.86 **
Ghrelin (pmol/L)	302.23 ± 18.85	269.56 ± 37.23 **
GABA (µmol/L)	0.51 ± 0.11	0.29 ± 0.13 ***
Dopamine (pmol/L)	190.37 ± 17.82	108.81 ± 19.19 ***
Serotonin (pmol/L)	0.52 ± 0.15	0.37 ± 0.13 ***
Epinephrine (pmol/L)	245.93 ± 21.70	302.85 ± 10.48 ***

All values are the mean ± SD, ** *p* < 0.01, *** *p* < 0.001 compared to study group.

**Table 2:** Pearson’s correlation coefficients between different variables in the control population.

Variable	Age (Years)		BMI		Serotonin (µmol/L)		Ghrelin (pmol/L)		GABA (µmol/L)		Dopamine (pmol/L)		Melatonin (pmol/L)		Adrenaline (pmol/L)	
	*r*	*p*	*r*	*p*	*r*	*p*	*r*	*p*	*r*	*p*	*r*	*p*	*r*	*p*	*r*	*p*
Age (years)	1	--	0.219	0.415	−0.450	0.08	0.377	0.15	0.108	0.692	−0.394	0.131	0.583 *	0.018	−0.403	0.121
BMI	0.219	0.415	1	--	−0.270	0.311	0.259	0.333	0.019	0.943	0.223	0.407	0.455	0.077	−0.087	0.749
Serotonin (µmol/L)	−0.450	0.080	−0.270	0.311	1	--	−0.219	0.416	0.230	0.392	0.420	0.106	−0.242	0.366	0.298	0.262
Ghrelin (pmol/L)	0.377	0.150	0.259	0.333	−0.219	0.416	1	--	−0.089	0.743	−0.375	0.153	0.434	0.093	−0.115	0.671
GABA (µmol/L)	0.108	0.692	0.019	0.943	0.230	0.392	−0.089	0.743	1	--	−0.008	0.976	0.061	0.821	−0.183	0.497
Dopamine (pmol/L)	−0.394	0.131	0.223	0.407	0.420	0.106	−0.375	0.153	−0.008	0.976	1	--	−0.255	0.340	0.304	0.252
Melatonin (pmol/L)	0.583 *	0.018	0.455	0.077	−0.242	0.366	0.434	0.093	0.061	0.821	−0.255	0.340	1	--	−0.435	0.092
Adrenaline (pmol/L)	−0.403	0.121	−0.087	0.749	0.298	0.262	−0.115	0.671	−0.183	0.497	0.304	0.252	−0.435	0.092	1	--

* Correlation is significant at *p* < 0.05 (2-tailed).

**Table 3:** Pearson correlation coefficient between different variables in study population.

Variable	Age (Years)		BMI		Serotonin (µmol/L)		Ghrelin (pmol/L)		GABA (µmol/L)		Dopamine (pmol/L)		Melatonin (pmol/L)		Adrenaline (pmol/L)	
	*r*	*p*	*r*	*p*	*r*	*p*	*r*	*p*	*r*	*p*	*r*	*p*	*r*	*p*	*r*	*p*
Age (years)	1	--	−0.178	0.510	0.247	0.356	−0.096	0.723	0.270	0.312	−0.280	0.294	−0.032	0.906	0.218	0.418
BMI	−0.178	0.510	1	--	0.202	0.452	0.584 *	0.017	−0.009	0.973	0.167	0.535	−0.067	0.807	−0.027	0.921
Serotonin (µmol/L)	0.247	0.356	0.202	0.452	1	--	−0.038	0.888	0.340	0.198	0.150	0.580	−0.148	0.583	0.181	0.503
Ghrelin (pmol/L)	−0.096	0.723	0.584 *	0.017	0.038	0.888	1	--	0.031	0.908	0.087	0.748	0.111	0.682	−0.271	0.311
GABA (µmol/L)	0.270	0.312	−0.009	0.973	0.340	0.198	0.031	0.908	1	--	−0.156	0.564	−0.520 *	0.39	0.304	0.252
Dopamine (pmol/L)	−0.280	0.294	0.167	0.535	0.150	0.580	0.087	0.748	−0.156	0.564	1	--	0.186	0.489	−0.285	285
Melatonin (pmol/L)	−0.032	0.906	−0.067	0.807	−0.148	0.583	0.111	0.682	−0.520 *	0.039	0.186	0.489	1	--	0.165	0.541
Adrenaline (pmol/L)	0.218	0.418	−0.027	0.921	0.181	0.503	−0.271	0.311	0.304	0.252	0.285	0.285	0.165	0.541	1	--

* Correlation is significant at *p* < 0.05 (2-tailed).

**Table 4:** Difference in BMI and serum biomarkers in estrogen receptor-positive and estrogen receptor-negative male breast cancer patients.

Serum Biomarker	Estrogen Receptor-Positive (*n* = 10)	Estrogen Receptor-Negative (*n* = 6)
BMI	23.84 ± 1.83	23.76 ± 2.09
Serotonin (μmol/L)	0.38 ± 0.15	0.36 ± 0.12
Ghrelin (pmol/L)	269.9 ± 40.17	270.13 ± 35.55
GABA (μmol/L)	0.31 ± 0.11	0.26 ± 0.10
Dopamine (pmol/L)	111.51 ± 21.32	104.30 ± 18.25
Melatonin (pmol/L)	62.72 ± 8.99	59.20 ± 8.56
Epinephrine (pmol/L)	302.54 ± 10.39	303.38 ± 11.63

All values are mean ± SD; there was no significant difference.

## Data Availability

Data is contained within the article.
